# Predicting fear and perceived health during the COVID-19 pandemic using machine learning: A cross-national longitudinal study

**DOI:** 10.1371/journal.pone.0247997

**Published:** 2021-03-11

**Authors:** Stephanie Josephine Eder, David Steyrl, Michal Mikolaj Stefanczyk, Michał Pieniak, Judit Martínez Molina, Ondra Pešout, Jakub Binter, Patrick Smela, Frank Scharnowski, Andrew A. Nicholson

**Affiliations:** 1 Department of Cognition, Emotion, and Methods in Psychology, Faculty of Psychology, University of Vienna, Vienna, Austria; 2 Department of Psychiatry, Psychotherapy and Psychosomatics, Psychiatric Hospital, University of Zürich, Zürich, Switzerland; 3 Institute of Psychology, University of Wroclaw, Wroclaw, Poland; 4 Faculty of Psychology, University of Barcelona, Barcelona, Spain; 5 Department of Psychology, Jan Evangelista Purkyne University, Ústí nad Labem, Czech Republic; 6 Department of Anthropology, Faculty of Humanities, Charles University, Prague, Czech Republic; 7 Neuroscience Center Zürich, University of Zürich and Swiss Federal Institute of Technology, Zürich, Switzerland; 8 Zürich Center for Integrative Human Physiology (ZIHP), University of Zürich, Zürich, Switzerland; 9 Department of Psychiatry and Behavioural Neurosciences, McMaster University, Hamilton, Canada; 10 Homewood Research Institute, Guelph, Canada; Middlesex University, UNITED KINGDOM

## Abstract

During medical pandemics, protective behaviors need to be motivated by effective communication, where finding predictors of fear and perceived health is of critical importance. The varying trajectories of the COVID-19 pandemic in different countries afford the opportunity to assess the unique influence of ‘macro-level’ environmental factors and ‘micro-level’ psychological variables on both fear and perceived health. Here, we investigate predictors of fear and perceived health using machine learning as lockdown restrictions in response to the COVID-19 pandemic were introduced in Austria, Spain, Poland and Czech Republic. Over a seven-week period, 533 participants completed weekly self-report surveys which measured the target variables subjective fear of the virus and perceived health, in addition to potential predictive variables related to psychological factors, social factors, perceived vulnerability to disease (PVD), and economic circumstances. Viral spread, mortality and governmental responses were further included in the analysis as potential environmental predictors. Results revealed that our models could accurately predict fear of the virus (accounting for approximately 23% of the variance) using predictive factors such as worrying about shortages in food supplies and perceived vulnerability to disease (PVD), where interestingly, environmental factors such as spread of the virus and governmental restrictions did not contribute to this prediction. Furthermore, our results revealed that perceived health could be predicted using PVD, physical exercise, attachment anxiety and age as input features, albeit with smaller effect sizes. Taken together, our results emphasize the importance of ‘micro-level’ psychological factors, as opposed to ‘macro-level’ environmental factors, when predicting fear and perceived health, and offer a starting point for more extensive research on the influences of pathogen threat and governmental restrictions on the psychology of fear and health.

## Introduction

### Fear and the crisis

The global Coronavirus Disease 2019 (COVID-19) pandemic affected millions of people and forced the mobilization of governments worldwide. New regulations were adopted around the globe, and responses from citizens to novel measures were diverse [1]. Importantly, individual responses are critical in shaping the course of the current pandemic and of comparable health crises, since modern-day human behavior greatly influences the propagation and extinction of diseases (e.g., by following hygiene recommendations, stay at home orders, practicing physical distancing, and achieving immunity through vaccinations) [2]. A critical factor in understanding a population’s response to a threat is the fear it elicits, since fear is an important predictor of behavioral changes and health-securing behaviors [3–6]. Thus, fear appeal is one of the most effective interventions to control health-related pandemics via behavioral changes, where accurate estimations of fear levels in a given population are essential for informing decisions with respect to educational and preventive interventions [7]. Theoretical frameworks such as protection motivation theory and the health belief model have been conceptualized to predict health-related behaviors in relation to mechanisms of fear appeal [8, 9]. They highlight that perceived *threat*, perceived *vulnerability/susceptibility* and perceived *efficacy*/*benefits and barriers* are all critical components for promoting protective behaviors; importantly, these models have been used widely to design behavioral interventions [8, 9]. Indeed, studies drawing upon these theoretical frameworks have confirmed the predictive power of these variables with regard to health-related behaviors during the COVID-19 crisis [10–13]. In relation, it has been shown that *perceived* risk for oneself–as opposed to actual risk–partially accounts for compliance with rules [14]. Critically, this compliance interacts with interpersonal, ‘micro-level’ variables (i.e., individual variables such as biological sex, age, and personality traits) in combination with macro-level variables such as governmental rules [14, 15].

Indeed, some individuals perceive situations as more threatening than others, and whilst many studies on the efficacy of fear appeals have emerged, less is known about which factors actually influence how much fear a threat elicits and how this interacts with individual differences. This is of critical importance for public health campaigns that need to effectively communicate the magnitude of a threat in order to guide health promoting behaviors. Interestingly, manipulation checks in laboratory fear-appeal studies tend to only find moderate associations between fear induction and induced fear [6]. It is therefore of theoretical interest and practical importance when designing health policies to consider variables that predict the subjective fear of the Sars-CoV-2 virus in a real-life setting. In response, we aim to identify both macro-level environmental and micro-level psychological variable that can specifically predict subjective fear of the COVID-19 virus.

Factors influencing fear and behavioral changes in the face of threats may be perceived vulnerability to diseases [16], both generally and specific to COVID -19 [11, 17], and aversion to germs, a concept related to disgust sensitivity, which has been shown to be implicated in subjective fear of COVID-19 [18]. Further, social support [19] and close relationships may buffer the fear-inducing effects of an external threat, since they are crucial for well-being and health [20]. In relation, attachment style influences affect regulation [21], and insecure attachment has been linked to heightened sensitivity for anxiety [22], as well as personal fear of death [23]. Traditionally, attachment theory explains behaviors and attitudes towards a close person in stressful situations [24]. As such, it provides a useful framework for investigating individual reactions to environmental stressors (such as the medical and economic threats elicited by a pandemic) and social stressors (such as the withdrawal of social contacts resulting from governmental restrictions). For example, individuals with high attachment security may be able to draw upon more optimal and adaptive psychological resources in order to effectively cope with such stressors related to COVID-19 [25]. Critically, secure attachment relates to an internal locus of control [26], which in turn has been shown to be a protective factor against the stress elicited by the COVID-19 pandemic [27].

Finally, objective environmental conditions, such as death tolls, might also affect fear levels; however, it remains unclear how objective measures of threat interact with psychological variables in the context of this unprecedented pandemic. Uncovering the variables that influence this relationship between external threats and the extent of fear elicited by these threats might guide targeted policies that avoid overreactions (i.e., panic purchases), while conversely, eliciting a sense of threat that is sufficient to motivate compliance with protective public health recommendations (i.e., social distancing and wearing masks in public settings). This study aims to shed light on the predictive value of environmental conditions, perceived vulnerability to disease, social factors and attachment security, in predicting fear of the novel coronavirus during the COVID-19 pandemic. As such, it aims to provide guidance for effectively constructing public health strategies and identifying target groups for behavioral interventions.

### Social isolation and impact on perceived health

Globally, social isolation and the shut-down of all public life has been essential to containing the spread of the virus [28]. Citizens were asked to stay at home and to avoid anyone other than the people they live with, which was enforced with fines in some countries [29]. For people living alone, this isolation may be particularly painful, as lockdown restrictions and curfews limit the possibilities of social interaction. Social contact can be ensured over social media, but the deprivation of physical contact has negative social and health implications, since touch plays an important role in maintaining and stabilizing social relations and provides various health benefits (such as reducing anxiety) that would be valuable in the face of a stressful crisis [30–32]. Overall, increases in loneliness, anxiety, and depression are likely [33] and have been reported in the context of this crisis [34, 35].

These changes likely have an impact on a populations’ perceived health, since psychological well-being and loneliness in turn predicts physical and perceived health [36, 37]. Lower perceived health has indeed been reported during the COVID-19 pandemic [38], and is associated with high levels of stress during this crisis [39]. Importantly, perceived vulnerability to the disease may also mediate such relationships between perceived health and emotional reactions related to fear and stress [40]. It may further interact with social consequences of the crisis, since loneliness resulting from social isolation has been found to partially mediate the effects of perceived vulnerability to COVID-19 on traumatic stress related to the pandemic [17].

A protective factor against the negative impacts of this crisis on perceived health may similarly be secure attachment, where various studies have linked secure attachment to more optimal coping mechanisms and positive health outcomes [41, 42]. It has been suggested that the benefits of physical interpersonal interactions are particularly valuable for people high in attachment anxiety [43]. A special case of physical interaction, sexual behavior, has also been linked to greater health outcomes [44], but is likely to decrease for singles or those living alone as curfews and lockdown restrictions are implemented. Further, physical activity positively influences perceived health and well-being [45], and has similarly been highly restricted during lockdowns. Here, we aim to investigate which factors predict perceived health throughout the crisis, and explore the complex and dynamic role of physical contact, attachment, sexual behavior, perceived vulnerability to disease, exercising, and various environmental conditions related to the COVID-19 pandemic. In doing so, we hope to identify protective factors that may help to maintain a high perceived state of health, which might mitigate excessive stress despite external medical and economical threats and social isolation.

### The current study

Taken together, the goal of the current study was to cross-culturally predict i) fear of the virus and ii) perceived health, as social isolation measures in response to the COVID-19 pandemic were being both enforced and dissolved within Europe. Here, fear was self-assessed and operationalized as the subjective fear and threat perceived by participants, both to themselves and to people that are emotionally close to them, of being harmed and/or becoming infected by the virus; whereas subjective health was the self-assessed overall perceived health state of participants.

By incorporating both features specific to the individual (e.g., perceived vulnerability and attachment style) and their environment (e.g., spread of the virus, governmental restrictions, living situation, economic threats), we utilize machine learning to predict fear of the virus and perceived health status. We then identify the variables that contribute most to these predictions. Employing machine learning as opposed to conventional analyses presents the advantage of predicting, rather than just explaining, psychological outcomes, in addition to clearly identifying useful predictors [46, 47]. The utility of machine learning models with regards to understanding complex human states and behavior has been repeatedly demonstrated in recent years [27, 48]. More specifically, these models represent a very robust way of multivariate data analysis, which can incorporate and control for a large amount of input variables while avoiding overfitting [49].

We hypothesize that our machine learning models will predict accurately reported fear levels when a variety of input factors are taken into account and controlled; specifically, we expect that perceived vulnerability to disease, attachment security and environmental factors will have high predictive value. Similarly, we hypothesize that machine learning models will be able to predict accurately perceived health, when utilizing information pertaining to living situations, exercising, sexual behavior, touching interactions, and perceived vulnerability to the pandemic threat as input variables.

Importantly, we aim to elucidate the factors that contribute most to the predictions of fear and perceived health. These variables may then help to target specific groups for behavioral interventions including fear appeal, and to design tailored interventions to counteract a decrease in perceived health as lockdown restrictions in response to this global threat are implemented.

## Methods

### Participants

Our sample consisted of 533 adult participants (mean age = 30.48, SD = 12.18), the majority of which were female (n = 345 female). Individuals participated in our study over a 7-week period, allowing us to collect repeated measurements of both static and changing environmental and psychological conditions, which served as inputs into our machine learning models. The amount of surveys completed by each participant varied, where some elected to take part every week, and some only filled in one or two questionnaires (three questionnaires on average, n = 1639 surveys in total). The study comprised of participants from the following countries: Austria (n = 190), Poland (n = 136), Spain (n = 107) and Czech Republic (n = 56). An additional 43 participants residing in other countries (Germany, United Kingdom, Ireland, Italy, and Pakistan) filled in surveys.

Additionally, 68% of participants were in a committed romantic relationship at the time of the study, and only 9% of all participants reported living alone during stay-at-home restrictions. The majority of participants had not yet experienced a COVID-19 case in their immediate social sphere and did not throughout the whole study period, although this differed markedly between the nations (82% in Austria, 94% in Poland, 57% in Spain, 91% in Czech Republic). Furthermore, 60% of participants reported to have experienced some economic disadvantages due to the crisis, but only 17% were certain that this could prove to be an existential threat.

No exclusion criteria were applied. All cases with at least one missing variable of interest were excluded from the machine learning models (see Results for trials included in each model). This sample is the same as investigated in other projects: https://osf.io/db4px/.

### Procedure

For the duration of seven weeks, weekly surveys were administered via e-mail to participants who had been recruited over social media (convenience sample). The surveys were administered over the platform SoSci Survey (www.soscisurvey.com). Weekly responses were matched by a self-generated participant code. All participants were informed about the aim of study and that they could stop participating at any point. Participants were fully debriefed and received the option to leave a contact address to be informed of the results of the study. Communication with the participants took place in their native language. All procedures of the data collection were performed in accordance with the GDPR regulation for data handling and the 1964 Helsinki Declaration and later amendments. We received ethical approval by the Institutional Review Board of Charles University as part of a larger program of research.

### Timeframe and political situation

Administration of our surveys occurred during a phase when all observed countries had recently implemented various measures to fight the spread of the virus. The first questionnaire was sent out to all participating countries during the week of March 16^th^ to 22^nd^, 2020, the last one was to be completed during the week of April 27^th^ to May 3^rd^, 2020.

**Fig 1:**
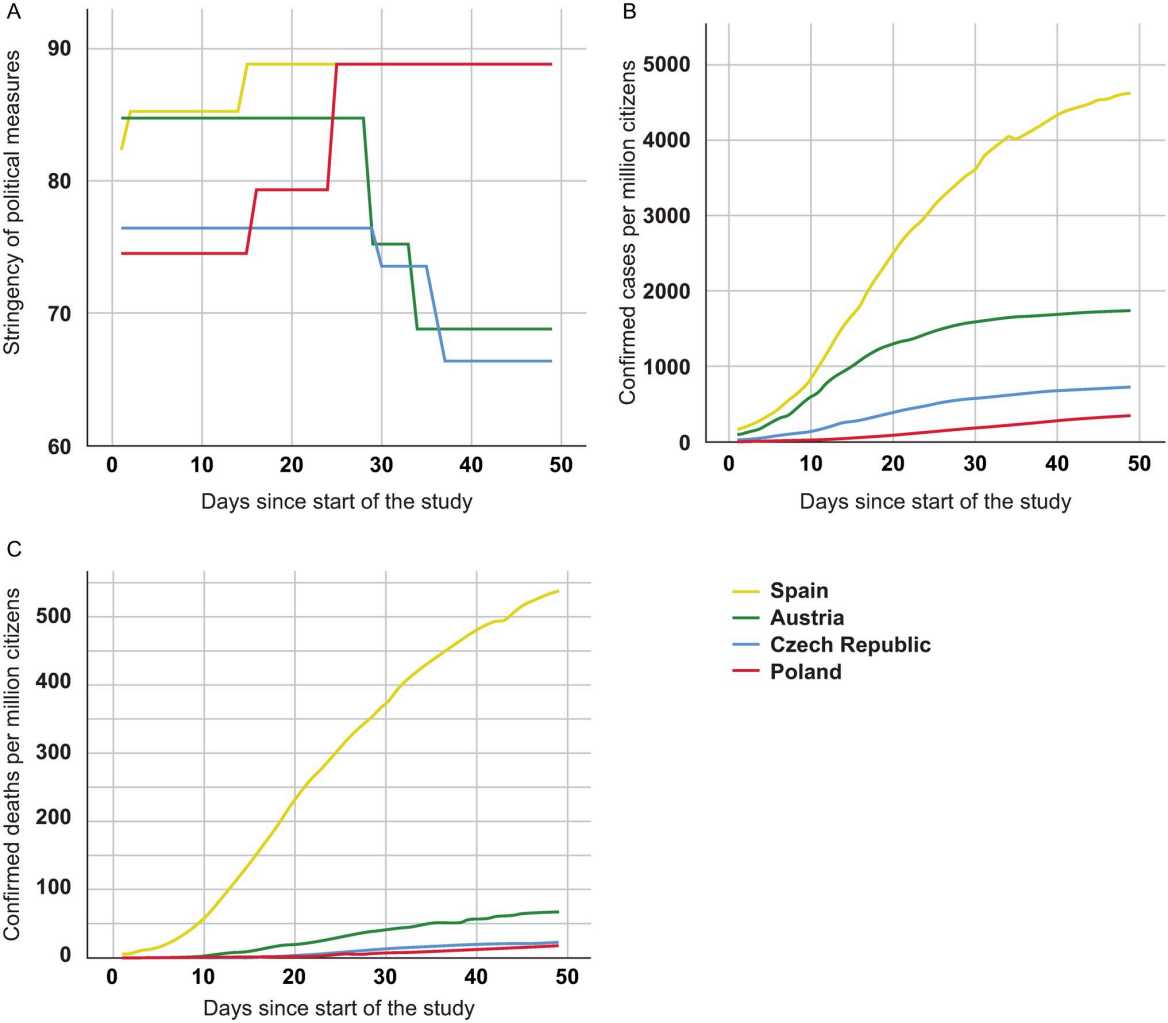
Spread of COVID-19 and governmental reactions. (A) Severity of governmental measures in response to the pandemic summarized as a stringency index (SI) [28], (B) confirmed cases and (C) deaths per million citizens caused by COVID-19 over the course of the study for Spain (yellow), Austria (green), Poland (red) and Czech Republic (blue). Source: Hale et al. [50]; Eurostat [51].

The actual spread and effects of the virus, as well as the defensive measures taken to counteract the virus, differed between the observed countries (Fig 1). Importantly, this range allowed us to optimally examine the predictive validity of these key features on fear and perceived health. Petherick and colleagues [28] summarize the gravity of governments’ responses to the COVID-19 crisis as a stringency index, based on indicators of ‘containment and closure policies’, ‘economic policies’ and ‘health system policies’. Fig 1 shows how this index developed, as well as confirmed cases and confirmed deaths per million citizens over the period of observation in each country. Notably, these numbers are influenced by the testing and reporting policies in each country.

### Surveys

Participants were asked to fill in the Perceived Vulnerability to Disease Scale (PVD, [52]) and the Experiences in Close Relationships Revised (ECR-R, [53]; Polish version: [54]; Czech version: [55]; Spanish version modified from: [56]) twice, around four weeks apart, in order to ensure stability of these constructs. The PVD is used as a measure of participants’ general perceived vulnerability to infectious diseases and their aversion towards actions that could potentially cause them, whereas the ECR-R is a self-report measure of adult attachment style that is widely used in research studies examining inner representations of social relationships (e.g. [57]). The PVD scale includes the subscales infectability (Cronbach’s α = .925) and germ aversion (Cronbach’s α = .705); the ECR-R consists of the subscales anxiety (Cronbach’s α = .920) and avoidance (Cronbach’s α = .917), where securely attached individuals would typically score low on both these scales [53]. If available, validated translations of the questionnaires were used, otherwise the questionnaire was translated by a native speaker and reviewed by another native speaker. The translations of the PVD to German, Spanish, Polish and Czech including values regarding their internal consistency and temporal stability are available on OSF: https://osf.io/2a4rc/. Additionally, each week we assessed i) information regarding sexual behaviour, ii) asked how much physical contact to other persons the participant had, ii) how much the participant exercised in the last week, iv) if the political measures taken could prove to be economically threatening to the participant, and v) how satisfied the participant was with the measures taken by their government. Further, we asked i) how the participant would assess their health state (target variable ‘health’, 5-point Likert-scale), and ii) about their fear of infection, fear that the virus might threaten their own health, and fear that the virus would threaten the health of people emotionally close to them (target variable ‘fear of the virus’, 5-point Likert scale, the three questions were averaged).

Demographic and personal information were additionally collected, including biological sex, sexual orientation, relationship status, living situation, weight, height, and country of residence (for a summary of all variables see Table 1). For each week and country, we included the aforementioned stringency index describing the severity of governmental measures in response to the COVID-19 crisis as calculated by Petherick et al. [28, Oxford COVID-19 Government Response Tracker] based on indicators such as travel bans, workplace closing and stay at home requirements. We further include confirmed COVID-19 cases and deaths per million citizens (weekly) as indicators of the current viral spread and mortality in each country (Sources: [50, 51], see Fig 1).

**Table 1:** Features included in machine learning models to predict the targets ‘fear’ and ‘perceived health’.

Target	Fear of the Virus	Perceived Health
*Macro-level environmental features*	Local and temporal spread of SARS-CoV-2 and mortality of COVID-19	Local and temporal spread of SARS-CoV-2 and mortality of COVID-19
	Stringency of lockdown restrictions	Stringency of lockdown restrictions
	(Country)	(Country)
*Demographic features*	Biological sex	Biological sex
	Sex. Orientation	Sex. Orientation
	Age	Age+
	Relationship status	(Relationship status)
	(Having children)	(Having children)
	Living situation (alone/with others)	Living situation (alone/with others)
	-	Weight/height
*Immediate personal effects of the crisis and response to the new situation*	Leaving house for occupational reasons	Leaving house for occupational reasons
	(Loss of income)	(Loss of income)
	(Existentially threatening economical loss)	Existentially threatening economical loss
	Infection in social sphere+	-
	Satisfaction with government decisions	(Feeling of preparedness)
	(Worry of outbreak and its consequences)+	(Worry of outbreak and its consequences)+
	Worry of food shortage+	(Worry of food shortage)+
	-	Exercise/week+
	(Exercise: actual vs. habit)	Exercise: actual vs. habit
*Psychological and social features*	Perceived health	Mean fear
	PVD+	PVD+
	ECR-R	ECR-R+
	Sexual activity	Sexual activity
	(Masturbation)	(Masturbation)
	(Levels of sexual arousal)	Levels of sexual arousal
	(Sexual satisfaction)	Sexual satisfaction
	Physical contact	Physical contact

*Note*. Features that were only included in the extensive models are set in parenthesis here. Features with a median proportional predictive value (permutation feature importance) greater than 0.05 in the better performing models are marked with ‘+’.

### Analysis

For each of the predicted variables (i.e., fear and health), we fit two types of machine learning models, one being a linear model (LASSO [least absolute shrinkage and selection operator], [58]), and one being a non-linear model (ERT [Extremely Randomized Trees], [59]). Analyses were conducted in Python 3.7.7. (Scikit-learn 0.22.2. [60]) and R [61]. The models were evaluated with a nested cross-validation procedure (90/10, 100 repeats each) [62]. Cross-validation allows one to assess model performance on new data, hence capturing the generalizability of the models [49]. Hyper-parameter tuning and feature scaling (z-scoring) took place in an inner loop within the main loop, using training data of the current cross-validation loop only. Categorical features were one-hot encoded [63]. Critically, these models allow us to incorporate many input features while avoiding over-fitting [49]. Extremely Randomized Trees further have the advantage of accurately capturing complex, non-linear relationships between different variables. The models were trained on repeated measurements from each participant, allowing for a good estimation of the impact of changing variables (e.g., governmental restrictions and mortality). Importantly, however, cross-validation was stratified, controlling for participant ID in order to counteract subject cluster learning. This procedure allows one to estimate the models’ performance in new, ‘unknown’ participants.

We compared this performance to a *trivial predictor*, which uses the mean of all target variables for each prediction. To estimate the predictive value of the input features, we report the median *permutation feature importance* (PFI) for the better-performing model as the proportional loss of explained variance if the input is replaced by a random (non-informative) array of that variable [64]. Initially, the models included all collected variables (see Table 1). Subsequently, we repeated the calculations with reduced input factors, taking into account only variables that were either influential (i.e., positive permutation feature importance) or explicitly expected to be influential (i.e. not just possible confounders) (Table 1). Since all trials with missing values in the input features were excluded, reducing the input dimensions leads to higher data density.

## Results

### Fear of the virus

Our machine learning models were able to predict mean fear of the virus significantly better than a trivial predictor. Specifically, the extensive models predict around 35% of the variance of fear ratings (LASSO: R^2^_avg_ = .35, R^2^_median_ = .35, *p*< .001; ERT: R^2^_avg_ = .32, R^2^_median_ = .36, *p*< .001; N_trials_ = 896), where the input ‘worrying about the outbreak and its consequences’ had the highest predictive value. Since this input is conceptually close to the target, we repeated the calculation without this input, and further reduced it by some input factors that evidenced lower predictive value (Table 1). The reduced models predicted approximately 23% of the variance, where again the linear models performed better (LASSO: R^2^_avg_ = .23, R^2^_median_ = .24, *p*< .001; N_trials_ = 1033; ERT: R^2^_avg_ = .22, R^2^_median_ = .22, *p*< .001; N_trials_ = 1033).

**Fig 2:**
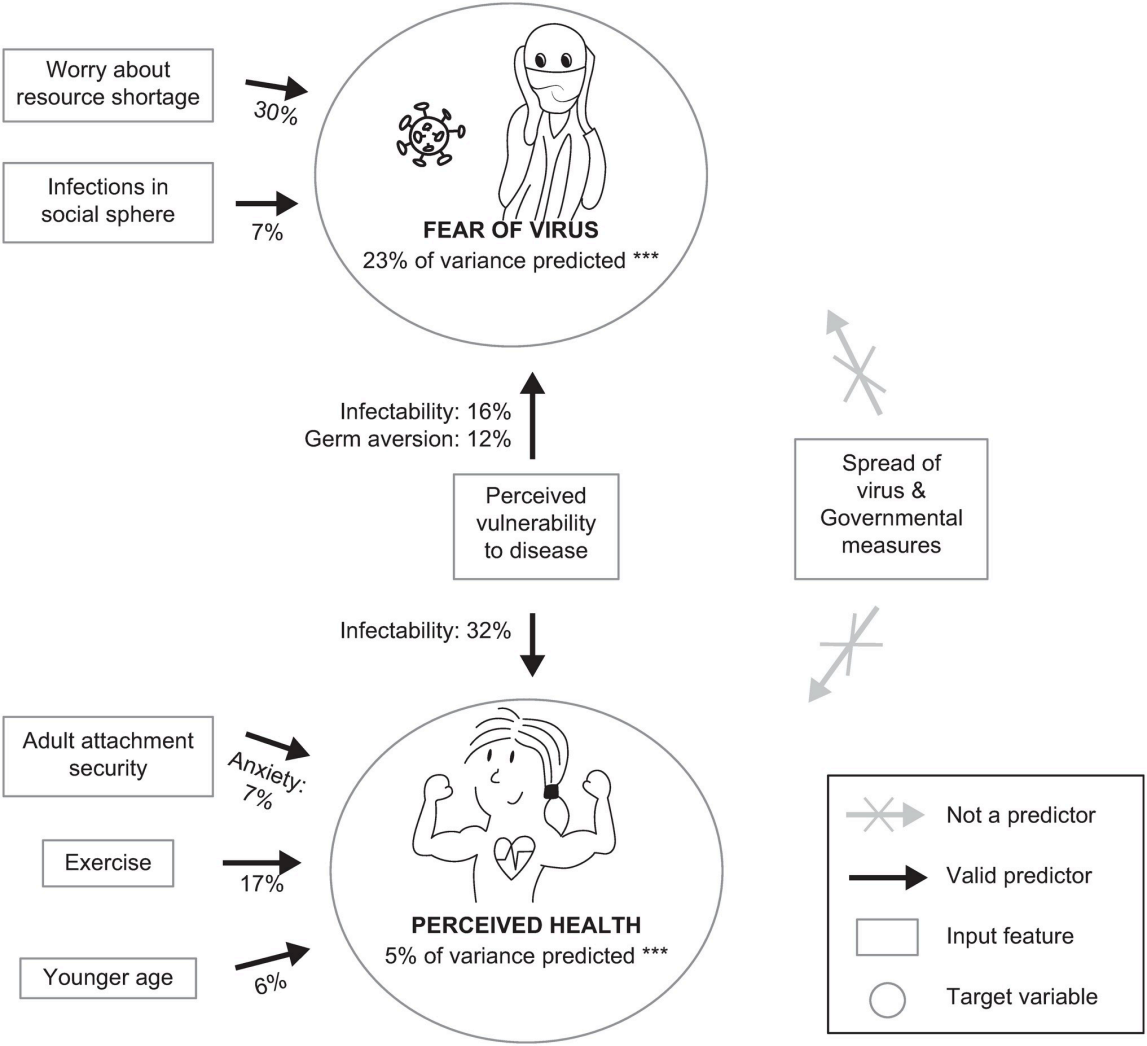
Summary of the results from the machine-learning models. The most important predictors of the better-performing models and the most notable variables with no predictive value are featured.

The most important predictors which contributed more than 5% to the overall explained variance of the variable ‘fear’ were i) worrying about food shortage (30.45%), ii) PVD scores (infectability: 16.33%; germ aversion: 12.21%), and iii) infections in the participant’s social sphere (7.08%), which all positively influenced fear ratings (Fig 2).

### Perceived health

Even though perceived health during the lockdown could be predicted significantly better by both our extensive and reduced models than by a trivial predictor, this is of little practical relevance since on average only 9% of the variance could be predicted with the reduced linear models (R^2^_avg_ = .09, R^2^_median_ = .13, *p*< .001, N_trials_ = 932), and 5% with the non-linear model (R^2^_avg_ = .05, R^2^_median_ = .09, *p*< .001, N_trials_ = 932). The more extensive models performed slightly worse, where on average, 5% of the variance was predicted (LASSO: R^2^_avg_ = .05, R^2^_median_ = .08, *p* = .002, N_trials_ = 896; ERT: R^2^_avg_ = .05, R^2^_median_ = .09, *p* = .003, N_trials_ = 896).

The most important predictors were the i) PVD score (infectability: 32.21%; higher scores predicting lower health ratings), ii) exercising (17.5%; more exercise/week predicting higher health ratings), iii) attachment security (anxiety: 6.67%; higher anxiety predicting lower health ratings) and iv) age (6.2%; younger age predicting higher health ratings). Sexual activity, physical contact, case counts and other country characteristics did not or only marginally improved the predictive value of the models (Fig 2).

## Discussion

The present study aimed to identify factors that could predict interpersonal differences in fear of the virus and perceived health as social isolation measures are implemented during the COVID-19 pandemic. We repeatedly administered questionnaires in four European states and trained machine-learning models to predict the outcome variables fear and perceived health. Our results revealed that fear of the virus could indeed be predicted with high accuracy (accounting for approximately 23% of the variance). Here, worries about resource shortage, perceived vulnerability to disease in general (PVD) [52], and infections in the participant’s social sphere were the most important predictors. Perceived health could be predicted better than by a trivial predictor, albeit at low effect sizes, where PVD, exercise, attachment security and age had some predictive value. Interestingly, macro-level environmental variables such as nationality, local and temporal spread of the virus, mortality, and the local stringency of governmental measures did not add predictive value during machine learning computations (Fig 2). Indeed, these results show the importance of considering ‘micro-level’ psychological variables over ‘macro-level’ environmental variables when predicting reactions to the COVID-19 pandemic (cf. [15, 65]). Here, we show the role of perceived vulnerability to diseases in general (as assessed by the PVD) in predicting fear of the COVID-19 threat, but also as a risk factor for experiencing poor subjective health during lockdown restrictions. Similarly, our results revealed that increased age and lower attachment security predicted lower perceived health; in other words, these individuals were at a higher risk for feeling unhealthy during these exceptionally stressful circumstances.

### Predicting fear

In the current cross-national machine learning study, approximately 23% of the variance of mean self-reported fear levels of the virus causing COVID-19 could be *predicted* based on variables such as ‘worrying about food shortages’, PVD (both infectability and germ aversion [52]), and ‘infections in the participant’s social sphere’. These results highlight that inter-individual factors significantly contribute to a population’s fears of the novel coronavirus (Sars-CoV-2), and support previous work calling attention to the ‘audience’ when investigating and designing behavioral interventions based on fear appeal [5]. Such studies have oftentimes reported sex differences with regard to this dimension [see 5]; interestingly, we found that other variables were much more influential predictors independent of reported biological sex (only 2% permutation feature importance).

Since fear of infection is an adaptive response to an environmental threat [66], we expected that it would be additionally influenced by environmental changes. Critically, however, our findings underscore that micro-level interpersonal, rather than macro-level environmental factors, contribute to fear development, since model prediction did not profit from input features related to objective pathogenic threat and governmental responses. This lack of influence on model prediction accuracy may reflect individual inabilities to process and understand the meaning of high digit numbers, such as the 2525.5 COVID-19 cases per million citizens communicated by the third week of the study in Spain, resulting in decreased capacities to rationally process the information (singularity effect, [67]). Additionally, the spread of misinformation with regard to the dangers of COVID-19 may have influenced how these numbers are processed (see [68]). Importantly, our findings emphasize the need for communication that is tailored to human psychology in order to help facilitate protective behaviors in times of crisis (see [69]). In line with this, it has been shown that fear arousal is an efficient promoter of disease-avoiding actions on the individual level [3, 4]. Indeed, an important theoretical framework for understanding fear appeals is protection motivation theory, which delineates appraisal processes that significantly influence changes in health-related behaviors. Threat appraisal is conceptualized as comprising of both the perceived severity of a threat and the perceived vulnerability to it, where coping appraisal is described as the perceived response to the threat in both efficacy and self-efficacy [8]. From the viewpoint of such frameworks, our results have elucidated aspects of threat appraisal related to the COVID-19 pandemic, which then need to be combined with efficacy-promoting messages (e.g. regarding the protective effects of face-masks and physical distancing) when used to define target groups and conceptualize behavioral interventions. Indeed, the importance of self-efficacy in fear appeal has been repeatedly demonstrated [6].

Importantly, fear induction alone will not solve a crisis, since fear-related behaviors may also contribute to harmful actions during viral pandemics [70], where Van Bavel et al. suggest that panic-driven behaviors (e.g., hoarding purchases) might contagiously be promoting individualistic behavior [71]. This is particularly dangerous when facing a threat where the most effective measures (e.g., wearing face masks) are aimed at protecting other people, not the individual. Stigmatization of infected individuals (both ‘victims and vectors’, [72]) could also be enhanced simultaneously with the fear of the virus [72], where vulnerable populations as well as populations at the front-line of fighting the deadly pandemic might inversely profit from fear-reducing interventions [73]. Taken together, an evidence-driven view by which psychological, demographic, and message-related [14] factors facilitate fear induction and behavioral changes in a given population is important to sensitize those who may play down a medical pandemic, while avoiding irrational, panic-driven behaviors.

### Predicting perceived health

In addition to the fear elicited by COVID-19, we aimed at predicting perceived health during this crisis, where we expected physical contact, sexual activity and environmental threats to have a high predictive value, which they did not. Nevertheless, perceived health could be predicted by our models significantly better than by a trivial predictor. Predictive factors for perceived health included not only exercising and perceived vulnerability to disease (both infectability and germ aversion), but also attachment security, thereby confirming previous findings [18, 40, 42]. Critically, however, the small effect size indicates that key variables influencing interpersonal differences in perceived health might have not been accurately captured in the current study. Future investigations should examine other potentially predictive features, which might include previously experiencing threatening illnesses [74], mindfulness [75, 76], and self-efficacy/individual locus of control [77].

### Limitations

It is unclear to which extent people with psychiatric disorders and medical conditions participated in the current study. Indeed, this may have distorting effects on perceived threat and health. Nevertheless, the cross-cultural composition should allow for some generalizations, in particular since our models are evaluated by their performance in predicting ‘unknown’ subjects and because nationality alone is not of predictive value for any of our target variables. Of course, this might change if more distant cultures were taken into account.

## Conclusion

The current cross-national longitudinal machine learning study provides critical insights with respect to predicting inter-individual differences in fear of the virus and perceived health during the COVID-19 pandemic in Europe. We identify predictors of both variables, where interestingly, psychological variables including perceived vulnerability to disease and attachment security were useful predictors, whereas macro-level environmental variables such as the local mortality rate and severity of lockdown restrictions had no predictive value. However, environmental variables directly affecting individuals, such as how much they exercised or infections within the participants’ immediate social sphere, did contribute to predicting our target variables.

Our models provide possible starting points for public communication strategies in order to facilitate appropriate behaviors that avoid and terminate health crises, and to support the people suffering most from stay-at-home requirements.

## References

[1] Jamison J, Bundy D, Jamison D, Spitz J, Verguet S. Comparing the impact on COVID-19 mortality of self-imposed behavior change and of government regulations across 13 countries. medRxiv. [Preprint]. 2020 [cited 2020 August 4]. www.medrxiv.org/content/10.1101/2020.08.02.20166793v1 10.1111/1475-6773.13688 PMC8441808 34182593

[2] Anderson RM, Heesterbeek H, Klinkenberg D, Hollingsworth TD. How will country-based mitigation measures influence the course of the COVID-19 epidemic? The Lancet. 2020; 395, 931–934. 10.1016/S0140-6736(20)30567-5 32164834 PMC7158572

[3] Green EC, Witte K. Can fear arousal in public health campaigns contribute to the decline of HIV prevalence?. Journal of health communication. 2006; 11(3), 245–259. 10.1080/10810730600613807 16624790

[4] Morman MT. The influence of fear appeals, message design, and masculinity on men’s motivation to perform the testicular self‐exam. Journal of Applied Communication Research. 2000; 28(2), 91–116. 10.1080/00909880009365558

[5] Tannenbaum MB, Hepler J, Zimmerman RS, Saul L, Jacobs S, Wilson K, et al. Appealing to fear: A meta-analysis of fear appeal effectiveness and theories. Psychological bulletin, 2015; 141(6), 1178. 10.1037/a0039729 26501228 PMC5789790

[6] Witte K, Allen M. A meta-analysis of fear appeals: Implications for effective public health campaigns. Health education & behavior. 2000; 27(5), 591–615. 10.1177/109019810002700506 11009129

[7] Pakpour A H, Griffiths M D. The fear of COVID-19 and its role in preventive behaviors. Journal of Concurrent Disorders. 2020; 2(1), 58–63. Available from: http://irep.ntu.ac.uk/id/eprint/39561 33195740

[8] Norman P, Boer H, Seydel ER. Protection motivation theory. In: Conner M, Norman P, editors. Predicting health behaviour. Open University Press. 2005; pp. 81–126.

[9] Champion VL, Skinner CS. The health belief model. In: Glanz K, Rimer BK, Viswanath K, editors. Health behavior and health education: Theory, research, and practice. 2008; pp. 45–65.

[10] Roma P, Monaro M, Muzi L, Colasanti M, Ricci E, Biondi S, et al. How to improve compliance with protective health measures during the covid-19 outbreak: testing a moderated mediation model and machine learning algorithms. International journal of environmental research and public health. 2020; 17(19), 7252. 10.3390/ijerph17197252 33020395 PMC7579153

[11] Prasetyo YT, Castillo AM, Salonga LJ, Sia JA, Seneta JA. Factors affecting perceived effectiveness of COVID-19 prevention measures among Filipinos during enhanced community quarantine in Luzon, Philippines: Integrating Protection Motivation Theory and extended Theory of Planned Behavior. International journal of infectious diseases; 2020. 99, 312–323. 10.1016/j.ijid.2020.07.074 32768695 PMC7406473

[12] Kowalski RM, Black KJ. Protection Motivation and the COVID-19 Virus. Health Communication. 2021; 36(1), 15–22. 10.1080/10410236.2020.1847448 33190547

[13] Wong LP, Alias H, Wong PF, Lee HY, AbuBakar S. The use of the health belief model to assess predictors of intent to receive the COVID-19 vaccine and willingness to pay. Human vaccines & immunotherapeutics. 2020; 16(9), 2204–2214. 10.1080/21645515.2020.1790279 32730103 PMC7553708

[14] Capraro V, Barcelo H. The effect of messaging and gender on intentions to wear a face covering to slow down COVID-19 transmission. arXiv:2005.05467 [Preprint]. 2020 [cited 2020 January 6th]. https://arxiv.org/ftp/arxiv/papers/2005/2005.05467.pdf

[15] Götz FM, Gvirtz A, Galinsky AD, Jachimowicz JM. How personality and policy predict pandemic behavior: Understanding sheltering-in-place in 55 countries at the onset of COVID-19. American Psychologist. 2020; 10.1037/amp0000740 33475389

[16] Weinstein ND. Perceived probability, perceived severity, and health-protective behavior. Health Psychology. 2000; 19(1), 65. 10.1037//0278-6133.19.1.65 10711589

[17] Boyraz G, Legros DN, Tigershtrom A. COVID-19 and traumatic stress: The role of perceived vulnerability, COVID-19-related worries, and social isolation. Journal of Anxiety Disorders. 2020; 76, 102307. 10.1016/j.janxdis.2020.102307 32937259 PMC7831572

[18] McKay D, Yang H, Elhai J, Asmundson G. Anxiety regarding contracting COVID-19 related to interoceptive anxiety sensations: The moderating role of disgust propensity and sensitivity. Journal of Anxiety Disorders. 2020; 102233. 10.1016/j.janxdis.2020.102233 32442880 PMC7194061

[19] Hornstein EA, Eisenberger NI. Unpacking the buffering effect of social support figures: social support attenuates fear acquisition. PloS one. 2017; 12(5), e0175891. 10.1371/journal.pone.0175891 28463999 PMC5413011

[20] Holt-Lunstad J, Smith TB, Layton JB. Social relationships and mortality risk: a meta-analytic review. PLoS medicine. 2010; 7(7), e1000316. 10.1371/journal.pmed.1000316 20668659 PMC2910600

[21] Marganska A, Gallagher M, Miranda R. Adult attachment, emotion dysregulation, and symptoms of depression and generalized anxiety disorder. American Journal of Orthopsychiatry. 2013; 83(1), 131–141. 10.1111/ajop.12001 23330631

[22] Watt MC., McWilliams LA, Campbell AG. Relations between anxiety sensitivity and attachment style dimensions. Journal of Psychopathology and Behavioral Assessment. 2005; 27(3), 191–200. 10.1007/s10862-005-0635-5

[23] Mikulincer M, Florian V, Tolmacz R. Attachment styles and fear of personal death: A case study of affect regulation. Journal of personality and social psychology. 1990; 58(2), 273–280. 10.1037/0022-3514.58.2.273

[24] Ainsworth M, Bowlby J. An ethological approach to personality development. American Psychologist. 1991; 46(4), 331–341. 10.1037/0003-066X.46.4.333

[25] Zakin G, Solomon Z, Neria Y. Hardiness, attachment style, and long term psychological distress among Israeli POWs and combat veterans. Personality and Individual Differences. 2003; 34(5), 819–829. 10.1016/S0191-8869(02)00073-9

[26] Hexel M. Alexithymia and attachment style in relation to locus of control. Personality and Individual Differences. 2003; 35(6), 1261–1270. 10.1016/S0191-8869(02)00333-1

[27] Flesia L, Monaro M, Mazza C, Fietta V, Colicino E, Segatto B, et al. Predicting Perceived Stress Related to the Covid-19 Outbreak through Stable Psychological Traits and Machine Learning Models. Journal of clinical medicine. 2020; 9(10), 3350. 10.3390/jcm9103350 33086558 PMC7603217

[28] Petherick A, Hale T, Phillips T, Webster S, et al. Variation in government responses to COVID-19. Blavatnik school working paper [Preprint] 2020 [cited 2020 May 8]. https://www.bsg.ox.ac.uk/research/publications/variation-government-responses-covid-19

[29] Govan F. What are the penalties for breaching Spain’s lockdown? And can you appeal? The Local. 2020 April 17 [Cited on May 20]. https://www.thelocal.es/20200417/how-much-can-you-be-fined-for-breaching-spains-lockdown-and-how-to-appeal

[30] Ditzen B, Neumann ID, Bodenmann G, von Dawans B, Turner RA, Ehlert U, et al. Effects of different kinds of couple interaction on cortisol and heart rate responses to stress in women. Psychoneuroendocrinology. 2007; 32(5), 565–574. 10.1016/j.psyneuen.2007.03.011 17499441

[31] Field T. Touch for socioemotional and physical well-being: A review. Developmental review, 2010; 30(4), 367–383. 10.1016/j.dr.2011.01.001

[32] Holt-Lunstad J, Birmingham WA, Light KC. Influence of a “warm touch” support enhancement intervention among married couples on ambulatory blood pressure, oxytocin, alpha amylase, and cortisol. Psychosomatic Medicine. 2008; 70(9), 976–985. 10.1097/PSY.0b013e318187aef7 18842740

[33] Brooks SK, Webster RK, Smith LE, Woodland L, Wessely S, Greenberg N, et al. The psychological impact of quarantine and how to reduce it: rapid review of the evidence. The Lancet. 2020; 395(10227), 912–920. 10.1016/S0140-6736(20)30460-8 32112714 PMC7158942

[34] Mazza C, Ricci E, Biondi S, Colasanti M, Ferracuti S, Napoli C, et al. Nationwide Survey of Psychological Distress among Italian People during the COVID-19 Pandemic: Immediate Psychological Responses and Associated Factors. Int. J. Environ. Res. Public Health. 2020; 17(9), 3165. 10.3390/ijerph17093165 32370116 PMC7246819

[35] Ahn S, Kim S, Koh K. Changes in Healthcare Utilization, Spending, and Perceived Health during COVID–19: A Longitudinal Study from Singapore [Preprint]. 2020 [cited 2020 January 15]. https://ssrn.com/abstract=3669090

[36] Kushlev K, Heintzelman SJ, Lutes LD, Wirtz D, Kanippayoor JM, Leitner D, et al. Does Happiness Improve Health? Evidence From a Randomized Controlled Trial. Psychological Science. 2020; 0956797620919673. 10.1177/0956797620919673 32579432

[37] Elran-Barak R, Mozeikov M. One month into the reinforcement of social distancing due to the COVID-19 outbreak: subjective health, health behaviors, and loneliness among people with chronic medical conditions. International journal of environmental research and public health. 2020; 17(15), 5403. 10.3390/ijerph17155403 32727103 PMC7432045

[38] Ohlbrecht H, Jellen J. Unequal tensions: the effects of the coronavirus pandemic in light of subjective health and social inequality dimensions in Germany. European Societies. 2021; 1–18. 10.1080/14616696.2020.1852440

[39] Szabo A, Ábel K, Boros S. Attitudes toward COVID-19 and stress levels in Hungary: Effects of age, perceived health status, and gender. Psychological Trauma: Theory, Research, Practice, and Policy. 2020; 12(6), 572–575.: 10.1037/tra0000665 32744843

[40] Inbar L, Shinan-Altman S. Emotional reactions and subjective health status during the COVID-19 pandemic in Israel: the mediating role of perceived susceptibility. Psychology, Health & Medicine. 2020; 1–10. 10.1080/13548506.2020.1858490 33315513

[41] Howard MS, Medway FJ. Adolescents’ attachment and coping with stress. Psychology in the Schools. 2004; 41(3), 391–402. 10.1002/pits.10167

[42] McWilliams LA, Bailey SJ. Associations between adult attachment ratings and health conditions: Evidence from the National Comorbidity Survey Replication. Health Psychology. 2010; 29(4), 446–453. 10.1037/a0020061 20658833

[43] Carmichael CL, Goldberg MH, Coyle MA. (2020). Security-Based Differences in Touch Behavior and Its Relational Benefits. Social Psychological and Personality Science. 2020. 10.1177/1948550619865057 32577160 PMC7310997

[44] Lindau ST, Gavrilova N. Sex, health, and years of sexually active life gained due to good health: evidence from two US population based cross sectional surveys of ageing. BMj. 2010; 340. 10.1136/bmj.c810 20215365 PMC2835854

[45] Piko B. Health-related predictors of self-perceived health in a student population: the importance of physical activity. Journal of Community Health. 2000; 25(2), 125–137. 10.1023/a:1005129707550 10794206

[46] Yarkoni T, Westfall J. Choosing prediction over explanation in psychology: Lessons from machine learning. Perspect. Psychol. Sci. 2017; 12, 1100–1122. 10.1177/1745691617693393 28841086 PMC6603289

[47] Orrù G, Monaro M, Conversano C, Gemignani A, Sartori G. Machine learning in psychometrics and psychological research. Front. Psychol. 2020; 10.3389/fpsyg.2019.02970 31998200 PMC6966768

[48] Dwyer DB, Efallkai P, Koutsouleris N. Machine learning approaches for clinical psychology and psychiatry. Annu. Rev. Clin. Psychol. 2018; 14, 91–118. 10.1146/annurev-clinpsy-032816-045037 29401044

[49] Cawley GC, Talbot NL. On over-fitting in model selection and subsequent selection bias in performance evaluation. The Journal of Machine Learning Research. 2010; 11, 2079–2107. Available from: www.jmlr.org/papers/volume11/cawley10a/cawley10a.pdf

[50] Hale T, Webster S, Phillips T, Kira K. Oxford COVID-19 Government Response Tracker, Blavatnik School of Government. 2020 [cited 2020 May 18]. https://www.bsg.ox.ac.uk/research/research-projects/coronavirus-government-response-tracker

[51] Eurostat. Population on 1 January; 2020 [cited 2020 April 23] (online data code:TPS00001). https://ec.europa.eu/eurostat/databrowser/view/tps00001/default/table?lang=en

[52] Duncan LA, Schaller M, Park JH. Perceived vulnerability to disease: Development and validation of a 15-item self-report instrument. Personality and Individual differences. 2009; 47(6), 541–546. 10.1016/j.paid.2009.05.001

[53] Fraley RC, Waller NG, Brennan KA. An item response theory analysis of self-report measures of adult attachment. Journal of personality and social psychology. 2000; 78(2), 350. 10.1037//0022-3514.78.2.350 10707340

[54] Lubiewska K, Gogowska K, Mickiewicz K, Wyrzykowska E, Winiewski C, Izdebski P, et al. Skala Experience in Close Relationships-Revised: Struktura, Rzetelno oraz Skrócona Wersja Skali w Polskiej Próbie. Psychologia Rozwojowa. 2016; 21 (1), 49–63. 10.4467/20843879PR.16.004.4793

[55] Cígler H, Cvrčková A, Daňsovác P, Hašto J, Charvát M, Ježek S, et al. Experiences in close relationships: České verze metod pro měření vazby vycházející z dotazníku ECR. E-psychologie. 2019; 13(4) 57–74. 10.29364/epsy.359

[56] Fernández-Fuertes AA, Orgaz B, Fuertes A, Carcedo R. La evaluación del apego romántico en adolescentes españoles: validación de la versión reducida del Experiences in Close Relationships-Revised (ECR-R). Anales de psicología. 2011; 27(3), 827–833. Available from: https://revistas.um.es/analesps/article/download/135561/123641/

[57] Goodall K, Trejnowska A. Darling S. The relationship between dispositional mindfulness, attachment security and emotion regulation. Personality and Individual Differences. 2012; 52(5), 622–626. 10.1016/j.paid.2011.12.008

[58] Tibshirani R. Regression shrinkage and selection via the lasso. Journal of the Royal Statistical Society: Series B (Methodological). 1996; 58(1), 267–288. Available from: https://www.jstor.org/stable/2346178

[59] Geurts P, Ernst D, Wehenkel L. Extremely randomized trees. Machine learning. 2006; 63(1), 3–42. 10.1007/s10994-006-6226-1

[60] Pedregosa F, Varoquaux G, Gramfort A, Michel V, Thirion B, Grisel O, et al. Scikit-learn: Machine learning in Python. The Journal of machine learning research, 2011; 12, 2825–2830. Available from http://jmlr.csail.mit.edu/papers/volume12/pedregosa11a/pedregosa11a.pdf

[61] R Core Team. R: A Language and Environment for Statistical Computing. Vienna: R Foundation for Statistical Computing; 2017. https://www.R-project.org

[62] Hastie T, Tibshirani R, Friedman J. The elements of statistical learning. Data Mining, Inference and Prediction. 2nd ed. New York: Springer; 2009.

[63] Potdar K, Pardawala TS, Pai CD. A comparative study of categorical variable encoding techniques for neural network classifiers. International journal of computer applications. 2017; 175(4), 7–9.

[64] Breiman L. Random Forests. Machine Learning. 2001; 45, 5–32. 10.1023/A:1010933404324

[65] Lawler EJ, Ridgeway C, Markovsky B. Structural social psychology and the micro-macro problem. Sociological Theory. 1993; 268–290. 10.2307/201971

[66] Schaller M, Park JH. The behavioral immune system (and why it matters). Current directions in psychological science. 2011; 20(2), 99–103. 10.1177/0963721411402596

[67] Dickert S, Västfjäll D, Mauro R, Slovic P. The feeling of risk: Implications for risk perception and communication. In: Cho H, Reimer T, McComas KA, editors. The SAGE handbook of risk communication. Thousand Oaks, CA, US: SAGE Publications; 2015. pp. 41–54.

[68] Pennycook G, McPhetres J, Zhang Y, Lu JG, Rand DG. Fighting COVID-19 Misinformation on Social Media: Experimental Evidence for a Scalable Accuracy-Nudge Intervention. Psychological Science. 2020. 10.1177/0956797620939054 32603243 PMC7366427

[69] Reissman DB, Watson PJ, Klomp RW, Tanielian TL, Prior SD. Pandemic influenza preparedness: adaptive responses to an evolving challenge. Journal of Homeland Security and Emergency Management. 2006; 3(2). 10.2202/1547-7355.1233

[70] Shultz JM, Cooper JL, Baingana F, Oquendo MA, Espinel Z, Althouse BM, et al. The role of fear-related behaviors in the 2013–2016 West Africa Ebola virus disease outbreak. Current psychiatry reports. 2016; 18(11), 104. 10.1007/s11920-016-0741-y 27739026 PMC5241909

[71] Van Bavel JJ, Baicker K, Boggio PS, Capraro V, Cichocka A, Cikara M, et al. Using social and behavioural science to support COVID-19 pandemic response. Nature Human Behaviour. 2020; 1–12. 10.1038/s41562-020-0818-9 32355299

[72] Pappas G, Kiriaze IJ, Giannakis P, Falagas ME. Psychosocial consequences of infectious diseases. Clin Microbiol Infect. 2009; 15:743–7. 10.1111/j.1469-0691.2009.02947.x 19754730 PMC7129378

[73] Ho SMY, Kwong-Lo RSY, Mak CWY, Wong JS. Fear of Severe Acute Respiratory Syndrome (SARS) Among Health Care Workers. Journal of Consulting and Clinical Psychology. 2005; 73(2), 344–349. 10.1037/0022-006X.73.2.344 15796643

[74] Thewes B, Butow P, Bell ML, Beith J, Stuart-Harris R, Grossi M, et al. Fear of cancer recurrence in young women with a history of early-stage breast cancer: a cross-sectional study of prevalence and association with health behaviours. Supportive Care in Cancer. 2012; 20(11), 2651–2659. 10.1007/s00520-011-1371-x 22328003

[75] Bränström R, Duncan LG, Moskowitz JT. The association between dispositional mindfulness, psychological well-being, and perceived health in a Swedish population-based sample. British journal of health psychology. 2011; 16(2), 300–316. 10.1348/135910710X501683 21489058 PMC3762484

[76] Roberts KC, Danoff-Burg S. Mindfulness and health behaviors: is paying attention good for you?. Journal of American college health, 2010; 59(3). 165–173. 10.1080/07448481.2010.484452 21186446

[77] Roddenberry A, Renk K. Locus of control and self-efficacy: potential mediators of stress, illness, and utilization of health services in college students. Child Psychiatry & Human Development. 2010; 41(4), 353–370. 10.1007/s10578-010-0173-6 20204497

